# Task-Specific Ionic Liquids with Lactate Anion Applied to Improve ZnO Dispersibility in the Ethylene-Propylene-Diene Elastomer

**DOI:** 10.3390/polym13050774

**Published:** 2021-03-03

**Authors:** Anna Sowińska, Magdalena Maciejewska, Laina Guo, Etienne Delebecq

**Affiliations:** 1 Institute of Polymer and Dye Technology, Lodz University of Technology, Stefanowskiego Street 12/16, 90-924 Lodz, Poland; 2 Hutchinson S.A-Research & Innovation Center, Rue Gustave Nourry BP31, 45120 Châlette sur Loing, France; laina.guo@hutchinson.com (L.G.); etienne.delebecq@hutchinson.com (E.D.)

**Keywords:** ionic liquids, task-specific ionic liquids, dispersion, zinc oxide, vulcanization

## Abstract

Task-specific ionic liquids (TSILs) are ionic liquids with structures and, consequently, properties and behaviors designed for particular applications. In this work, task-specific ILs with alkylammonium or benzalkonium cations and carboxyl groups in the form of lactate anions were used to promote the homogeneous dispersion of the curatives in the elastomer matrix. The reaction of carboxyl groups of TSILs with zinc oxide, which acts as a vulcanization activator, was confirmed. This interaction improved the solubility and dispersibility of zinc oxide particles in the ethylene-propylene-diene (EPDM) monomer matrix, which consequently affected the curing characteristics of rubber compounds. Most importantly, TSILs increased the efficiency of vulcanization by shortening the time, lowering the temperature and increasing the enthalpy of this process, while maintaining safe processing of elastomer composites. EPDM vulcanizates containing TSILs with lactate anion were characterized by satisfactory functional properties.

## 1. Introduction

The most common and general definition of ionic liquids (ILs) identifies these chemical compounds as salts with melting points lower than 100 °C. Another characteristic feature of ILs is the presence of a large, organic cation and an organic or inorganic anion in their structures. ILs are therefore compounds made entirely of ions [1,2]. Owing to their structure, ILs exhibit many unique properties—e.g., negligible vapor pressure, chemical and thermal stability, nonflammability, ionic conductivity and large electrochemical window [3,4,5,6]. Moreover, ILs possess a high solvation ability for a variety of organic polar and nonpolar chemicals or can form two-phase systems with many different solvents. Hence, ILs are increasingly used as environmentally friendly alternatives to common solvents for numerous applications [7,8], including various types of polymerization [9,10,11]. Increased research on the structure, properties and applications of ILs has been conducted for several years, but ILs are not new compounds. It is difficult to specify the beginning of the history of ILs and closely related molten salts, which are most commonly dated back to 1914 and based on the research of Walden on alkylammonium nitrates [12,13]. Reports on the first ILs appeared in the nineteenth century, when “red oil” was first received using Friedel–Crafts’ reaction. However, analytical methods to identify its structure were not available until the development of the Nuclear Magnetic Resonance (NMR) spectroscopy [14]. Nowadays, the cationic component of ILs varies from imidazolium cations to the pyrrolidinium, pyridinium, piperidinium, ammonium, thiazolium, phosphonium, benzalkonium and triazolium species. On the other hand, anions of ILs can be both organic (e.g., bis(trifluoromethylsulfonyl)imide) or inorganic (e.g., chloride, bromide, tetrafluoroborate or hexafluorophosphate). The huge variety of cations and anions that can be used to obtain ILs cause them to be tunable compounds, very often called “designer solvents” [15,16].

ILs are specific substances, owing to their structures being composed entirely of ions. Thus, they can be defined as pure electrolytes [17]. The technologically important properties of ILs result from strong electrostatic interactions that determine the fluid behavior. Although these electrostatic interactions are fairly strong, the size and nature of the cations very often make ILs liquids even at room temperature. However, at the microscopic level, their behaviors are pretty similar to the more conventional high temperature molten salts. The most widely used cations of ILs are those with *N*-heterocyclic rings. Imidazolium salts are the most significant in this group and most of them are commercially produced. Their properties such as solubility, melting temperatures and viscosity can be easily modified by varying the substituents at the nitrogen atoms of imidazole ring as well as by varying the counter-ions. Despite wide applications of imidazolium ILs in organic catalysis and synthesis, they have some defects. Imidazolium salts do not operate as neutral solvents under strongly alkaline environments, with deprotonation at carbon 2C resulting in the formation of *N*-heterocyclic carbenes [18]. Therefore, depending on the requirements, imidazolium ILs can be replaced with salts consisting of another cation.

The differences between the properties of classic solvents and ILs are significant. ILs have catalytic capabilities and can be optically active; additionally, their chirality is crucial during organic synthesis. The most preferred feature of ILs is their tunability. By varying the cations or anions, it is possible to design and obtain new ILs with specific and well defined properties, and thus intended for wide and specific applications. Consequently, in the last few years, the synthesis of ILs called “task-specific” (TSILs) has become increasingly popular. These specific properties of ILs result from the presence of appropriate functionalities in either cationic or anionic components of the ionic liquid. TSILs can often act both as reaction mediums and catalysts in some chemical transformations. These kinds of ILs still preserve the unique properties and can be easily recycled and reused as homogenous small molecular catalysts [19]. Since one of the most important applications of ILs is as reaction media for clean catalysis, Fei et al. developed a series of low-melting imidazolium salts bearing COOH groups. They described the synthesis and characterization of a series of ILs with carboxyl groups and melting temperatures lower than 61 °C [20]. Some researchers described lanthanide-containing TSILs as highly attractive for use as catalysts and promising soft luminescent materials [21]. The low solubility of lanthanide compounds hinders the possible applications of ILs, which require high amounts of dissolved metal to be applied to the electrodeposition of metals or as solvents for nanoparticle synthesis. Introducing lanthanide ions as an anion of ILs provides an encouraging way to enhance the concentration. An alternative way to improve the solubility of metal salts in ILs is application of TSILs, which possess a complexing moiety grafted onto the cation skeleton of ionic liquid. This method provides new opportunities for liquid–liquid extraction purposes. To overcome the problem of metal salts’ solubility, Nockemann et al. have developed TSILs with a functional carboxyl group that has the ability to coordinate with the metal ion. This facilitates the dissolution of metal oxides or metal salts in the presence of water [22,23]. When the functional group possesses the ability to coordinate to the metal ion (favorably as a bidentate or a polydentate ligand), it is easier to dissolve metal compounds such as metal salts or metal oxides in the ionic liquid. The carboxylic-functionalized ILs have the ability to dissolve huge amounts of metal oxides, such as zinc oxide (ZnO), so it seems to be interesting to use these ILs to enhance the solubility and the dispersion of ZnO in the elastomer matrix, especially since TSILs with carboxyl groups can be easily obtained and synthesized. There are many methods described in the literature. For example, Xiao et al. have synthesized Brønsted acidic ionic liquid, which was used as a catalyst in the coupling of carbon dioxide and epoxides to produce cyclic carbonates without in the absence of cosolvent [21].

ZnO is one of the main and most important components of elastomer composites, especially those cured with sulfur. Sulfur vulcanization is the most popular and commonly used industrial method for the crosslinking of unsaturated rubbers. Nevertheless, the chemistry and mechanism of sulfur crosslinking is very complicated and still quite incomprehensible. There is still no general agreement concerning the chemical nature of the active sulfurating agent and the mechanism of its reaction with the molecules of rubber, especially when ZnO is used as an activator [24]. Wolfe et al. [25] studied the effect of ZnO on the sulfur vulcanization mechanism using low molecular weight compounds as representative models of elastomers. The results showed a considerable influence of ZnO on the curing rate as well as the products of the crosslinking reactions. The curing rate increased and the product distribution changed, leading to the change in reaction mechanism. Many researchers suggested that active complexes of zinc with accelerators are characterized by higher reactivities than the free accelerator [26]. Coran has confirmed that the addition of ZnO as an activator has a pronounced effect on different steps of the vulcanization process [27,28]. It increases the rate of early scorch curing reactions that lead to the formation of the crosslink precursors and improves the extent of crosslinking, but decreases the rate of the actual vulcanization [27]. Regarding to this statement, Manik [29], Shelton [30] and Morita [31] et al. have concluded that the crosslinking reactions accelerated by sulfenamide systems are mixed radical and ionic in nature. When ZnO is present as an activator in the crosslinking system, it catalyzes the process of formation of the macromolecular complexes; thus, the amount of sulfur atoms, which are embedded in the accelerator, is enhanced due to the interaction of zinc ions with accelerators and the sulfur insertion occurs more rapidly. The zinc–accelerator complex is formed, which can be stabilized by ligands, which affect solubility of this complex in the rubber matrix [32]. Zinc atoms can coordinate with carboxylate ligands from stearic acid or with amine that is released from a sulfonamide; consequently, a significant improvement of solubility and reactivity of the zinc–accelerator complex is achieved. Thus, it is believed that TSILs with carboxyl groups can interact with ZnO or zinc–accelerator complexes similarly to stearic acid.

In this work, TSILs with alkylammonium or benzalkonium cations and carboxyl groups in the form of lactate anions were used to promote the homogeneous dispersion of the curatives in the ethylene-propylene-diene monomer (EPDM) matrix. Knowing that it is difficult to investigate the interaction between the components of the curing system inside the elastomer matrix, the interactions between ZnO and the ionic liquid during crosslinking were studied directly by preparing a mixture of these ingredients and then heating it to the temperature of vulcanization. Furthermore, the effect of TSILs on the rheometric properties of EPDM compounds and consequently on the crosslink density and mechanical properties of the vulcanizates were investigated.

## 2. Materials and Methods

### 2.1. Materials

**Scheme 1:**
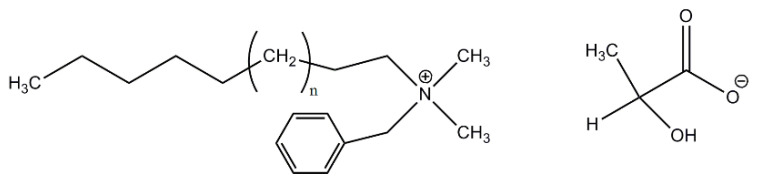
Structure of benzalkonium lactate (BALa).

**Scheme 2:**
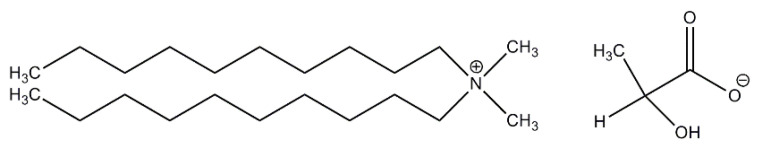
Structure of didecyldimethylammonium lactate (DDALa).

**Scheme 3:**
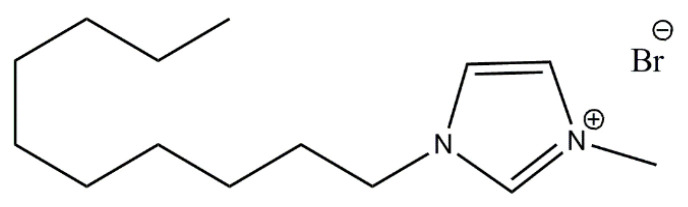
Structure of 1-decyl-3-methylimidazolium bromide (DmiBr).

An EPDM terpolymer consisting of 8.9% ethylidenenorbornene (ENB) and 58% ethylene was sourced from Exxon Mobil (Vistalon 8600, Irving, TX, USA). Its Mooney viscosity was ML1+4 (125 °C): 81. EPDM composites were filled with calcium carbonate (chalk, Omya BSH, Oftringen, Switzerland) and carbon black (CB, Spheron S0A; Cabot Corporation, Boston, MA, USA). Zinc oxide (ZnO, Huta Bedzin, Bedzin, Poland) was applied as a vulcanization activator. Mineral oil (Torillis 7200, Total Lubricants, Cedex, France) and stearic acid (POCH S.A., Gliwice, Poland) were applied as the processing oil and plasticizer, respectively. As the desiccant, calcium oxide granule (CaO, Kezadol GR, Kettlitz-Chemie, Rennertshofen, Germany) was used. Rubber compounds were cured using a conventional curing system with sulfur (Torimex-Chemicals, Lodz, Poland) as crosslinking agent, *N*-cyclohexyl-2-benzothiazolesulfenamide (CBS), zinc dialkylditiophosphate (Rhenogran TP50) and benzothiazole disulfide (MBTS) as accelerators, which were obtained from Brenntag Polska (Kedzierzyn-Kozle, Poland). Task-specific ionic liquids (TSILs) with lactate anions such as benzalkonium lactate (BALa) and didecyldimethylammonium lactate (DDALa) with the structures shown in Scheme 1 and Scheme 2 were synthesized by Industrial Chemistry Institute (Warsaw, Poland). 1-Decyl-3-methylimidazolium bromide (DmiBr) (purity > 98%), with the structure presented in Scheme 3, which does not possess the carboxyl functionality, was provided by IoLiTec Ionic Liquids Technologies GmbH (Heilbronn, Germany).

### 2.2. Characterization of TSILs and Possible Interaction between Their Carboxyl Groups and ZnO

The interaction between zinc oxide (ZnO) and carboxyl groups of TSILs was studied using the mixture of ZnO and the proper ionic liquid. DDALa, with the structural formula presented in Scheme 2, was chosen for this study. First, DDALa and ZnO were mixed directly in a glass laboratory beaker in a weight ratio of 1:1. Next, the mixture was heated at 70 °C for 30 min to form a homogeneous paste. For comparison, the same experiment was performed for a mixture of ZnO and DmiBr, which does not contain carboxyl group. This ionic liquid was chosen due to its considerable activity in the vulcanization, which was reported previously [33,34]. Prepared mixtures were marked as DDALa + ZnO and DmiBr + ZnO, respectively.

Thermogravimetric (TG) analysis was performed using a thermogravimetry/differential scanning calorimetry TGA/DSC1 analyzer (Mettler Toledo, Greifensee, Switzerland). Measurements were conducted in the temperature range of 25–600 °C to analyze the thermal decomposition of pure TSILs. Samples of TSILs with masses of 10 mg were heated in an inert atmosphere (argon, flow rate 50 mL/min) using a heating rate of 10 °C/min.

Differential scanning calorimeter DSC1 (Mettler Toledo, Greifensee, Switzerland), was used to study thermal transitions of pure TSILs, ZnO and their mixtures. The samples of pure TSILS were heated from the temperature −100 to 200 °C, with a heating rate of 10 °C/min, whereas measurements for pure ZnO and its mixtures with TSILs or DmiBr were performed in the temperature range of 25–180 °C to study the possible reactions in the range of vulcanization temperatures. Liquid nitrogen was used to cool the samples before and after the measurement. Nitrogen was used as a protective gas at the flow rate of 80 mL/min.

Fourier Transform Infrared (FTIR) spectroscopy was conducted using a FTIR Nicolet 6700 (Thermo Scientific Products: Riviera Beach, Florida, USA) spectrophotometer with OMNIC 3.2 software. FTIR spectra were collected in the range of wavenumbers from 4000 to 400 cm^−1^ over 128 scans. Attenuated Total Reflectance (ATR) equipped with a single reflection diamond ATR crystal on ZnSe plate was applied for all investigations.

Time-of-Flight Secondary Ion Mass Spectrometry was conducted using a TOF-SIMS IV (IONTOF GmbH, Münster, Germany) mass spectrometer. The instrument was equipped with a Bi liquid metal ion gun and mass resolution time of flight mass analyzer. The measuring area was 100 × 100 μm of the sample surface. The time of analysis was 30 s. During the analysis, the area of examined specimen was irradiated with the pulses of 25 keV Bi^3+^ ions at 10 kHz repetition rate and an average ion current of 0.4 pA. Mass spectra of positive and negative ions were recorded, corresponding to the products present in the mixtures tested.

Nuclear magnetic resonance (NMR) spectra were acquired using a Bruker Avance II Plus 16.4 T spectrometer (Bruker BioSpin, Rheinstetten, Germany) equipped with a 5 mm Z-gradient broadband decoupling inverse probe. The operating frequencies were 700 and 175 MHz for 1H and 13C experiments, respectively. The NMR sample was prepared by dissolving 10 mg of the compound in 600 μL of DMSO-d6 and the measurement was run at 300 K. The chemical shifts were referenced to the DMSO signal at 2.50 and 39.5 ppm. All spectral regions were individually corrected with a fifth-order baseline function.

### 2.3. Preparation and Characterization of EPDM Compounds

EPDM compounds were prepared using a two-step procedure as described in our previous work [34]. First, the masterbatch with the composition given in Table 1 was prepared using an internal mixer and then it was divided into four equal pieces and the curatives together with TSILs were added to each of these pieces using a laboratory two-roll mill. The general compositions of EPDM compounds containing TSILs are listed in Table 2. These rubber compounds were used to prepare the vulcanizates for which tests of mechanical properties, hardness and crosslink density were performed.

EPDM compounds were vulcanized at 150 °C with the optimal vulcanization time (t_95_), which was measured with the D-RPA 3000 rheometer (MonTech, Buchen, Germany). Based on the rheometric curves, the torque increase was determined using the following equation:

(1) $$\Delta S=S_{max}-S_{min},$$

where *S_max_* is the maximum of the torque during rheometric measurement (dNm) and *S_min_* is the minimum of the torque (dNm).

The density of crosslinks in EPDM vulcanizates was determined according to the ISO 1817 [35] standard, based on their equilibrium swelling in toluene at room temperature. The crosslink density was calculated with Flory–Rehner equation [36]. The Huggins parameter of elastomer–solvent (toluene) interactions, given by Equation (2) [37], was adopted for these calculations, where *V_r_* is the volume fraction of elastomer in swollen gel.

(2) $$\chi =0.425+0.340V_{r},$$

The range of EPDM vulcanization temperatures and enthalpy were analyzed by employing a differential scanning calorimeter DSC1 (Mettler Toledo, Greifensee, Switzerland). Measurements were performed for small pieces of rubber compounds with masses of approximately 10 mg. Differential scanning calorimetry (DSC) curves were recorded across a temperature range of −100 to 250 °C, with a heating rate of 10 °C/min.

Tensile tests of EPDM vulcanizates were carried out following the procedure included in the ISO 37 [38] standard using a Zwick Roell 1435 (Ulm, Germany) universal testing machine. The specimens had a standard Dumb-bell shape, which was characterized with test length of 20 mm and a width of 4 mm. The average thickness of the samples was 2 mm. Tensile tests were carried out at a crosshead speed of 500 mm/min at room temperature.

The hardness of disc-shaped EPDM vulcanizates was examined using a Shore A (Zwick/Roell) durometer (Ulm, Germany) according to the ISO 868 [39] standard.

Since the significant content of fillers and other ingredients, such as CaO or mineral oil, can make it difficult to investigate the distribution of curatives and ZnO in the EPDM matrix, additional rubber compounds were prepared with a simplified composition—i.e., containing only curing system and the proper ionic liquid (without fillers and other auxiliaries) in order to carefully examine the effect of TSILs on the distribution of curatives and ZnO in the crosslinked elastomer matrix. EPDM compounds with the simplified compositions given in Table 3 were prepared with a laboratory two-roll mill.

Scanning electron microscopy (SEM) analysis of EPDM vulcanizates was performed using an LEO1450 SEM microscope (Carl Zeiss AG, Oberkochen, Germany) in order to study the morphology and average size of ZnO and curatives particles, as well as their distribution in the elastomer matrix in the presence of TSILs. Before the measurements, vulcanizates were broken down after freezing in liquid nitrogen. Energy-dispersive X-ray spectroscopy (EDS) was employed to study the distribution of elements included in TSILs and ZnO in the crosslinked elastomer matrix. Prior to analysis, the fractures of samples were sputtered with carbon to raise the quality of SEM/EDS results.

## 3. Results

### 3.1. Thermal Stability of TSILs with Lactate Anione

**Figure 1:**
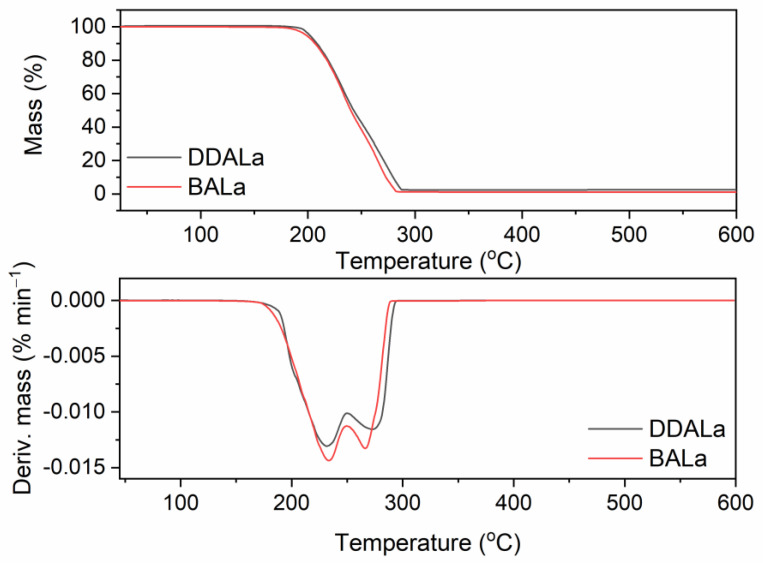
Thermogravimetric (TG) and Differential Thermogravimetric (DTG) curves for pure TSILs.

In the first step of the studies, thermogravimetric (TG) analysis was performed to characterize the thermal stability of TSILs and to determine their onset decomposition temperature. The results are presented in Figure 1.

T_5%_ was determined as the onset temperature of the thermal decomposition of TSILs. This parameter represents the temperature at which exactly 5% of the substance mass loss occurs relative to the initial mass of the sample. Regardless of the cation type, the studied TSILs exhibited similar thermal stabilities. Thermal decompositions of DDALa and BALa occurred in the temperature range of 180−300 °C and DDALa exhibited a slightly higher onset decomposition temperature than BALa (T_5%_ approximately 202 °C and 198 °C for DDALa and BALa, respectively). Most importantly, TG analysis confirmed that TSILs with lactate anions are thermally stable at the vulcanization temperature (150 °C) of rubber compounds and thus can be successfully used to support the vulcanization.

### 3.2. Phase Transition of TSILs with Lactate Anion

**Figure 2:**
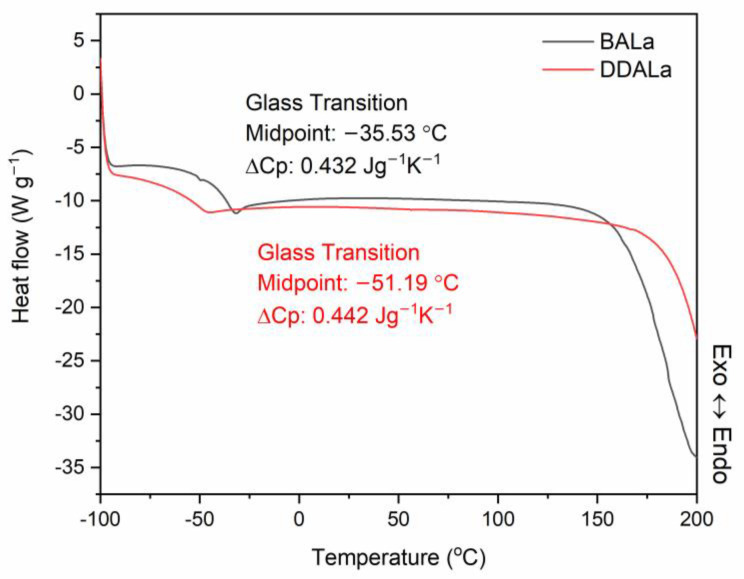
Differential scanning calorimetry (DSC) curves for pure DDALa and BALa.

**Figure 3:**
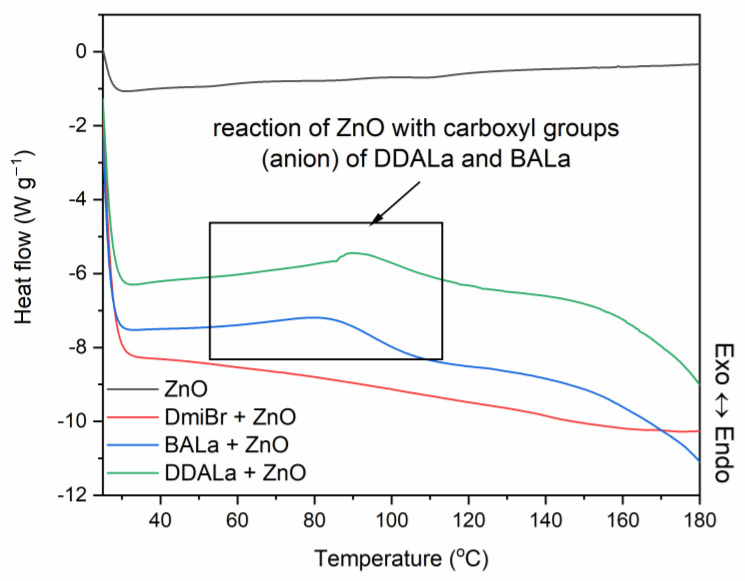
DSC curves for pure zinc oxide (ZnO) and its mixture with ILs.

In the next step of the research, the phase transitions of the pure TSILs with lactate anions were examined using DSC analysis. Furthermore, the mixtures of ZnO with DDALa and BALa were prepared with the weight ratio 1:1 and DSC measurements were performed for these mixtures to study the possible reaction/solubilization of ZnO in TSILs. In addition, DSC analysis was conducted for pure ZnO and its mixture with ionic liquid DmiBr, which does not bear a carboxyl group. DSC curves are presented in Figure 2 and Figure 3 and the results are summarized in Table 4.

From the analysis of the DSC curves presented in Figure 2, a glass transition was observed for both TSILs with T_g_ of approximately −35 °C for BALa and −51 °C for DDALa. The differences in T_g_ results from the structure of TSILs. Owing to the presence of the aromatic ring in the benzalkonium cation, BALa exhibited significantly higher T_g_ than DDALa with long didecyl chains in the ammonium cation. At temperatures above 160 °C for BALa and 180 °C for DDALa, the endothermic thermal decomposition of both TSILs began, which was confirmed by the results of TG analysis (Figure 1). No melting peaks were observed in the DSC curves, because studied TSILs are liquid at room temperature.

From the analysis of the DSC curve of pure ZnO, no thermal transitions were observed in the temperature range of 25–180 °C, whereas broad exothermic peaks were seen on DSC curves of ZnO mixtures with BALa and DDALa in a temperature range of 63−109 °C, with an enthalpy of approximately 4.0 J/g and a temperature of the peak of approximately 92 °C (Figure 3). On the other hand, no peak was observed in the DSC curve of the ZnO mixture with DmiBr. Therefore, the exothermic peaks on DSC curves of ZnO/TSILs mixtures were concluded to result from the reaction of ZnO with carboxyl group (anion) of DDALa and BALa. Consequently, these ILs can react with ZnO and improve the ZnO dispersion in the rubber matrix, resulting in better contact between ZnO particles and other components of the curing system, can be expected. Nockemann et al. have described that the TSILs bearing a carboxyl group have a selective solubilizing ability toward metal oxides in the presence of water [22,23,40]. Thus, SEM/EDS analysis was performed for the vulcanizates containing curatives, ZnO and TSILs with lactate anions to confirm this assumption. Moreover, additional techniques were employed to examine the preheated mixtures of ZnO and TSILs to confirm above conclusion.

### 3.3. FTIR Analysis of ZnO and Its Mixtures with ILs

**Figure 4:**
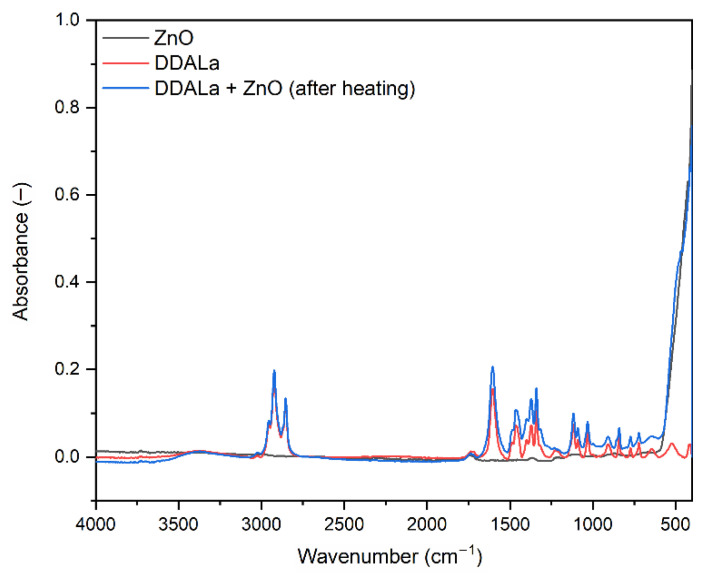
FTIR spectra for ZnO and DDALa mixture after heating at 70 °C for 30 min.

**Figure 5:**
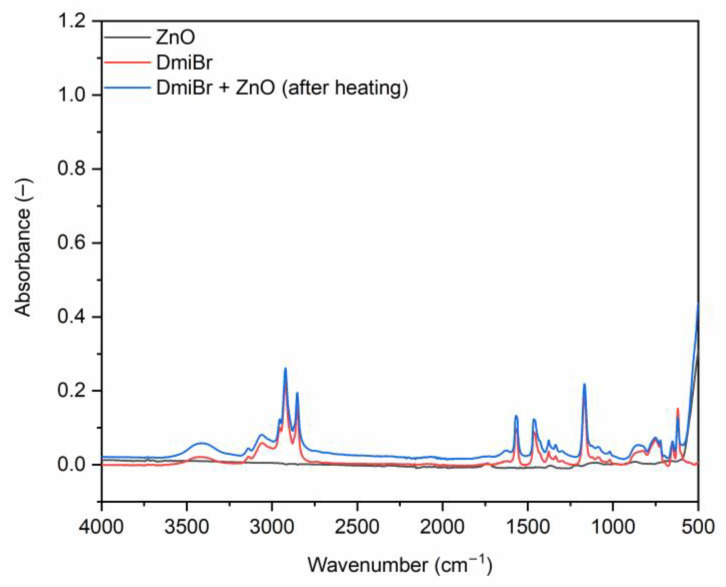
FTIR spectra for ZnO and DmiBr mixture after heating at 70 °C for 30 min.

**Figure 6:**
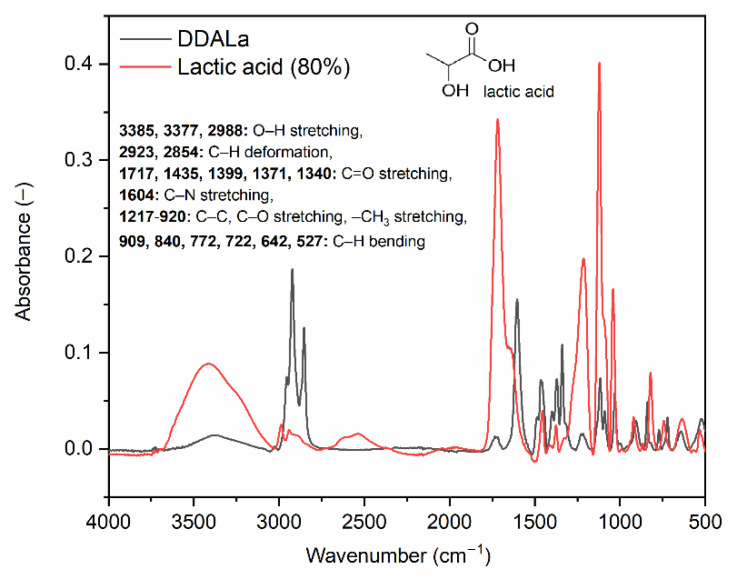
Fourier Transform Infrared Spectroscopy (FTIR) spectra for DDALa and lactic acid—identification of bands characteristic for lactate anion of DDALa.

FTIR analysis was employed to examine the preheated mixtures of ZnO and TSILs to confirm the postulated reaction of ZnO with carboxyl groups (anion). DDALa was chosen for this study. The measurement was also performed for the mixture of ZnO and ionic liquid DmiBr, which does not possess a carboxyl group. FTIR analysis was carried out for pure reagents (ZnO, DDALa and DmiBr) and for their mixture before and after heating at 70 °C for 30 min. Results are presented in Figure 4 and Figure 5. Additional measurement was carried out for pure lactic acid to identify which bands present in the FTIR spectrum of DDALa refer to lactate anion (carboxyl group) of this ionic liquid (Figure 6).

Regarding the FTIR spectra presented in Figure 6, the C=O stretching bands at 1717 1435, 1399, 1371 and 1339 cm^−1^, which are characteristic of the carboxyl group of lactic acid and lactate anions, were identified. The following bands characteristic for the O–H stretching vibrations of the lactic acid component were identified at 3377 and 2988 cm^−1^, while the bands in the region 1217–920 cm^−1^ correspond to stretching modes of C–C and C–O functional groups. The bands occurring at the wavenumbers below 910 cm^−1^ (909, 840, 772, 642 and 527 cm^−1^) generally refer to C–H vibrations (bending, deformation). The bands at 1116 and 1031 cm^−1^ correspond to –CH_3_ group and C–C bonds of lactate anions and in the case of DDALa also to C−C bonds of didecyl chains and –CH_3_ group of didecyldimethylammonium cations. Additional bands at 2923 and 2854 cm^−1^ in the FTIR spectrum of DDALa correspond to C−H bonds of didecyl chains, whereas the band at 1604 cm^−1^ refers to C−N bonds in the didecyldimethylammonium cation.

Analyzing the FTIR spectra of the DDALa and ZnO mixture (Figure 4), there are no significant differences in the position and intensity of the FTIR bands for the pure DDALa and its mixture with ZnO. Characteristic bands of lactate anion were identified at 2922, 2853, 1735, 1466, 1370, 1217 and 908 cm^−1^, respectively. Thus, FTIR spectroscopy confirmed that the reaction between ZnO and DDALa did not change the structure of the lactate anion of the ionic liquid. A highly intensive band in the range of 600−500 cm^−1^ confirmed the presence of zinc in the prepared mixture. Similar results were observed for the DmiBr and ZnO mixture (Figure 5). There are no differences in the FTIR spectra collected for the pure DmiBr and its mixture with ZnO, except for the band at approximately 500 cm^−1^, which corresponds to the Zn−O vibrations and confirms the presence of ZnO in the mixture. The intensities and positions of bands characteristic for DmiBr at 2954, 2852 cm^−1^, 1569, 1465, 1377, 1167, 749 and 651 cm^−1^, respectively, are almost identical in the spectrum of pure DmiBr and its mixture with ZnO. Thus, no reaction occurred between these reactants when the mixture was heated at 70 °C for 30min. On the other hand, FTIR analysis did not allowed for detection of the ionic interactions between ZnO and the lactate anion of DDALa. Therefore, TOF-SIMS spectroscopy was employed, which is suitable for detecting ionic structures.

### 3.4. TOF-SIMS Analysis of DDALa and ZnO Mixture

**Figure 7:**
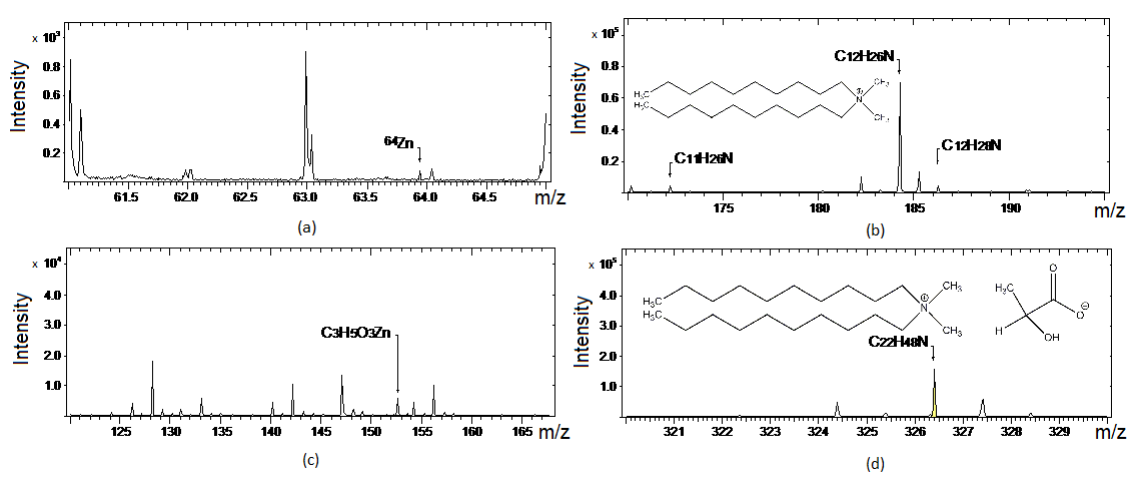
The secondary ion mass (TOF-SIMS) spectra for positive ions of DDALa and ZnO mixture: (**a**) Zn^2+^; (**b**) C_12_H_26_N^+^, C_12_H_28_N^+^; (**c**) C_3_H_5_O_3_Zn^+^; (**d**) C_22_H_48_N^+^.

**Figure 8:**
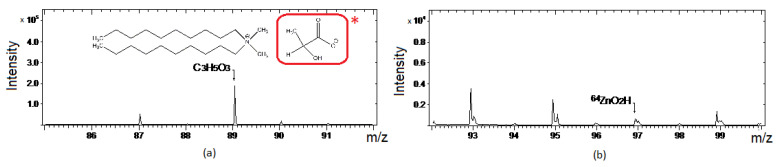
TOF-SIMS spectra for negative ions of DDALa and ZnO mixture: (**a**) C_3_H_5_O_3_^−^; (**b**) ZnO_2_H^−^; (* lactate anion of DDALa).

**Figure 9:**
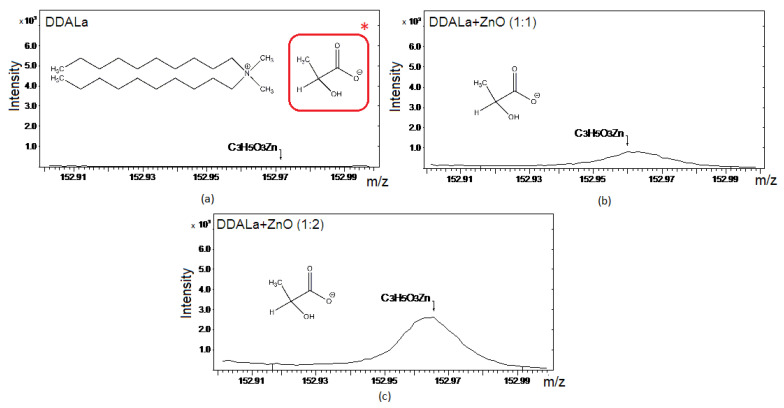
TOF-SIMS spectra for C_3_H_5_O_3_Zn^+^ ion recorded for: (**a**) pure DDALa; (**b**) **DDALa and ZnO mixture with the weight ratio of** 1:1; (**c**) DDALa and ZnO mixture with the weight ratio of 1:2; (* lactate anion of DDALa).

TOF-SIMS measurements were conducted for the mixtures of ZnO with DDALa with two different weight ratios, such as 1:1 and 1:2, respectively, in order to increase the intensity of the peaks and achieve more accurate results. Prior to the measurement, samples were placed on a quartz glass plate and then studied. The results are presented in Figure 7, Figure 8 and Figure 9.

TOF-SIMS analysis performed for the 1:1 DDALa and ZnO mixture allowed the detection of some *m*/*z* ratios of positive and negative ions that are characteristic for the components of the mixture (Figure 7 and Figure 8). Regarding the spectra of positive ions, *m*/*z* 63.9 corresponds to Zn^2+^ from zinc oxide (Figure 7a), whereas *m*/*z* 184.5, 189.0 and 326.4 refer to C_12_H_26_N^+^, C_12_H_28_N^+^ (Figure 7b) and C_22_H_48_N^+^ (Figure 7d) from the cation of DDALa, respectively. On the other hand, *m*/*z* 89 in the spectrum of negative ions corresponds to C_3_H_5_O^3−^ (Figure 8a), the lactate anion of DDALa, whereas *m*/*z* 97 refers to ZnO_2_H^−^ and confirmed the presence of ZnO in the tested mixture (Figure 8b). However, the most important is the peak for *m*/*z* 153 corresponding to the C_3_H_5_O_3_Zn^+^ ion, which was observed in the spectrum of positive ions (Figure 7c). Presence of this ion indicates the interaction/reaction between ZnO and lactate anion of DDALa. To confirm this conclusion, an additional TOF-SIMS measurement was performed for pure DDALa (Figure 9a) and its mixture with ZnO with the weight ratio of 1:2—i.e., with increased amount of zinc oxide (Figure 9c). As it was expected, the peak for *m*/*z* 153 referring to C_3_H_5_O_3_Zn^+^ ion was present in the TOF-SIMS spectrum collected for 1:2 DDALa and ZnO mixture, but its intensity was twice as large as for the 1:1 DDALa and ZnO mixture (Figure 9b). Moreover, the peak for *m*/*z* 153 did not occur in the spectrum of pure DDALa (Figure 9a). Therefore, it was proven that this peak resulted from reaction between ZnO and the lactate anion of DDALa.

### 3.5. Nuclear Magnetic Resonance (NMR) Analysis of ZnO and Its Mixtures with ILs

NMR spectra of pure DDALa and the mixture of DDALa and ZnO with the weight ratio 1:2 were collected by dissolving 10 mg of the studied compound in 600 μL of DMSO-d6. The same analysis was performed for pure DmiBr and its mixture with ZnO.

**Figure 10:**
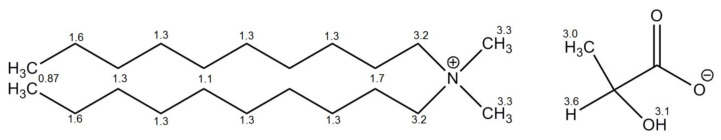
Structural formula of DDALa with marked protons.

**Figure 11:**
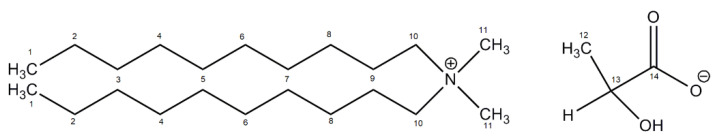
Structural formula of DDALa with marked carbons.

The structural formulas of DDALa with marked protons or carbons used in this analysis are presented in Figure 10 and Figure 11.

**Figure 12:**
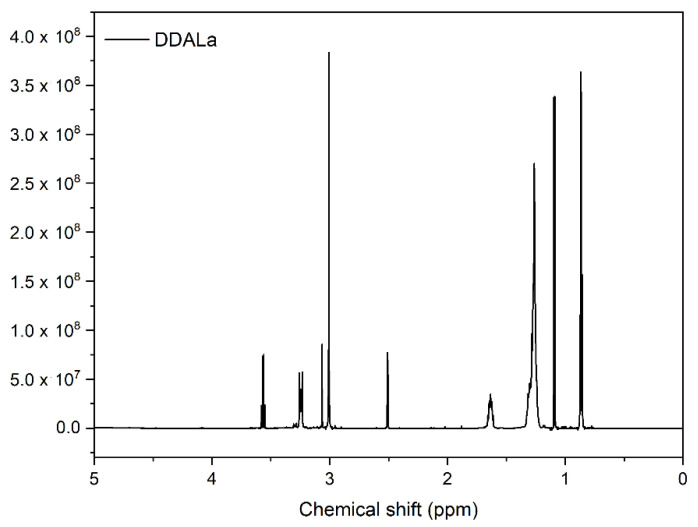
^1^H Nuclear Magnetic Resonance (NMR) spectrum for pure DDALa.

**Figure 13:**
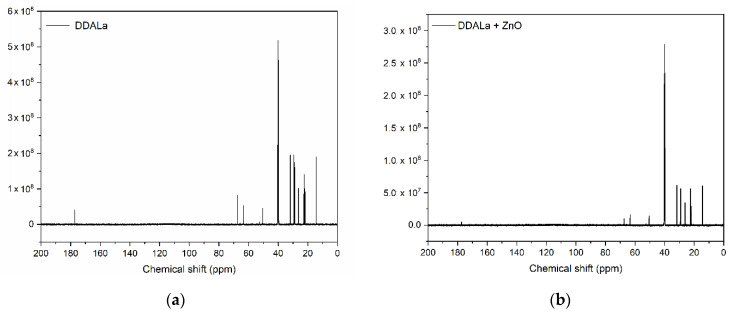
^13^C NMR spectra: (**a**) pure DDALa; (**b**) 1:2 mixture of DDALa and ZnO.

**Figure 14:**
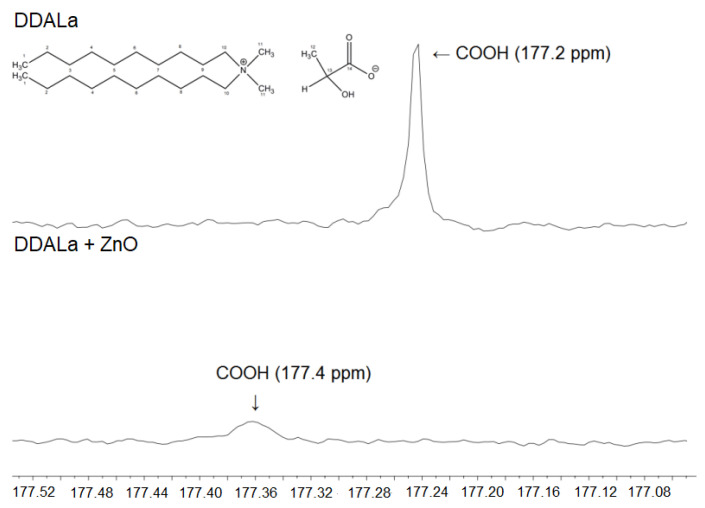
^13^C NMR spectra for 1:2 DDALa and ZnO mixture—identification of interactions.

The NMR spectra for pure DDALa and its mixture with ZnO are presented in Figure 12, Figure 13 and Figure 14.

^1^H NMR spectra are presented only for pure DDALa (Figure 12). Measurements of the mixture of DDALa and ZnO were not performed because after analyzing the results of FTIR and TOF-SIMS, it was concluded that the surroundings of the hydrogen atoms of DDALa in the mixture did not change compared to pure DDALa. Regarding the ^1^H NMR spectra of pure DDALa, the chemical shifts characteristic for DDALa were identified, as follows: ^1^H NMR (DMSO), *s—singlet, d—double, t—triplet: 0.87 (–CH_3_, t, 6H), 1.1 (–CH_2_, d, 4H), 1.3 (–CH_2_, t, 16H), 1.6 (–CH_2_, s, 4H), 1.7 (–CH_2_, d, 4H), 2.5 (DMSO), 3.0 (–CH_3_, s, 3H), 3.3 (–OH, s, 1H), 3.2 (–CH_2_, d, 4H), 3.3 (–CH_3_, d, 6H) and 3.6 (–CH, s, 1H).

^13^C NMR analysis had a crucial role in this part of the study—it allowed for identification of peaks corresponding to specific carbon atoms present in the DDALa molecule and matched them to the structure of DDALa. Results are presented in Figure 13 and Figure 14 and in Table 5.

Comparing the ^13^C NMR spectrum of pure ionic liquid and its mixture with ZnO, a difference in the chemical shift corresponding to the carbon of the carboxyl group (C14) was observed (177.2 and 177.4 ppm for pure DDALa and its mixture with ZnO, respectively), as can be seen in Figure 14. In addition, the intensity of this peak in the spectrum of the mixture was much smaller than for pure DDALa. This indicates that surroundings of the carboxyl group were changed, e.g., due to the reaction of the lactate anion with ZnO. The difference in the chemical shift of C14 in pure DDALa and its mixture with ZnO was not so considerable, but it should be noted that the chemical shifts of other carbons in the pure DDALa and its mixture with ZnO were the same, and no changes were detected.

**Figure 15:**
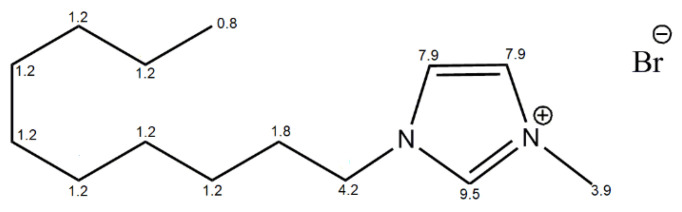
Structural formula of DmiBr with marked protons.

**Figure 16:**
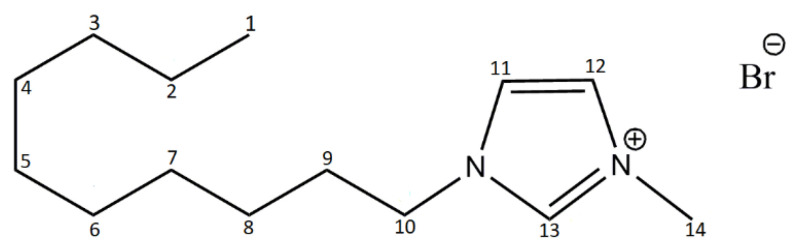
Structural formula of DmiBr with marked carbons.

The structural formulas of DmiBr with marked protons and carbons used in this analysis are presented in Figure 15 and Figure 16.

**Figure 17:**
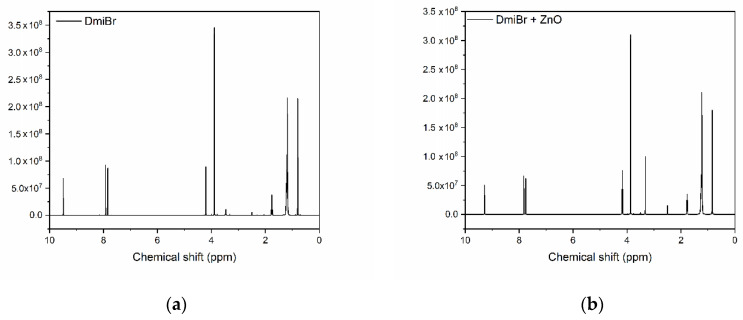
^1^H NMR spectra: (**a**) pure DmiBr; (**b**) 1:1 mixture of DmiBr and ZnO.

**Figure 18:**
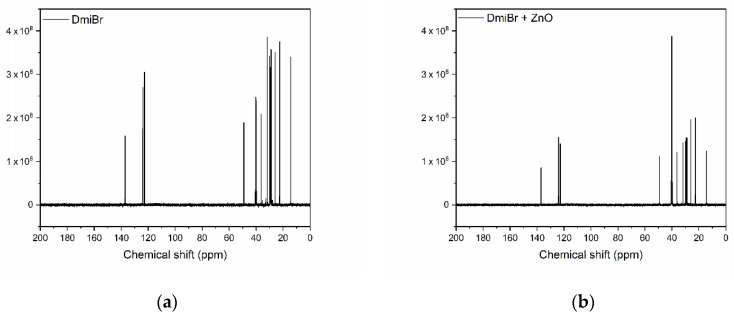
^13^C NMR spectra: (**a**) pure DmiBr; (**b**) 1:2 mixture of DmiBr and ZnO.

As far as the mixture of DmiBr and ZnO is concerned, the results of ^1^H NMR and ^13^C NMR analysis are presented in Figure 17 and Figure 18 and in Table 6.

^1^H NMR spectra for pure DmiBr and 1:1 mixture of DmiBr and ZnO are presented in Figure 17. Measurements were performed to verify if any interaction between ZnO and DmiBr occurs and thus if the surroundings of hydrogen or carbon atoms in the DmiBr structure changes accordingly. From analysis of the ^1^H NMR spectra, no differences between pure DmiBr (Figure 17a) and its mixture with ZnO (Figure 17b) were detected, and the intensities of the peaks were also almost identical. The only exception was the peak with the chemical shift 3.3 ppm obtained for the DmiBr and ZnO mixture, the intensity of which increased as compared to that of the pure DmiBr. However, this peak corresponds to hydroxyl group of DMSO solvent and did not result from any interaction between DmiBr and ZnO [41]. Regarding the ^1^H NMR spectra of pure DmiBr and its mixture with ZnO, the chemical shifts were identified as follows: ^1^H NMR (DMSO), *s—singlet, d—double, t—triplet, m—multiplet: 0.8 (–CH_3_, t, 3H), 1.2 (–CH_2_, m, 14H), 1.8 (–CH_2_, m, 2H), 2.5 (DMSO), 3.3 (–OH, s, 1H), 3.9 (–CH_3_, s, 3H), 4.2 (–CH_2_, t, 2H), 7.9 (–CH, t, 2H) and 9.5 (–CH, s, 1H).

Comparing the ^13^C NMR spectrum of pure DmiBr and its mixture with ZnO, no changes in the chemical shifts were observed (Figure 18, Table 6), so the surroundings of carbon atoms in the DmiBr structure did not change when the mixture was heated at 70 °C for 30 min. Thus, any reaction between DmiBr and ZnO was identified using NMR analysis.

### 3.6. Effect of TSILs on Dispersion of ZnO and Curatives in the EPDM Matrix

**Figure 19:**
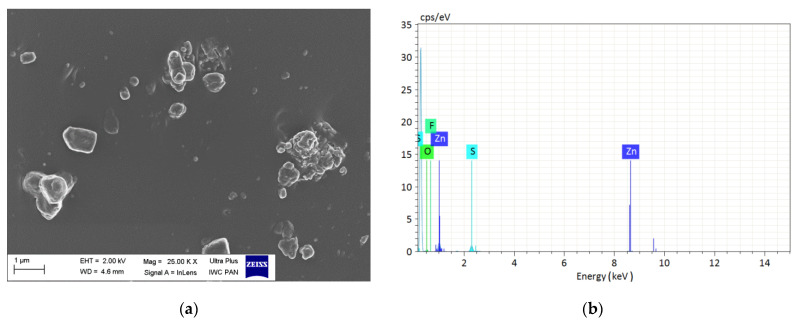
Dispersion of curatives in EPDM vulcanizate without ionic liquid: (**a**) Scanning electron microscopy (SEM) image; (**b**) Energy-dispersive X-ray spectroscopy (EDS) analysis.

**Figure 20:**
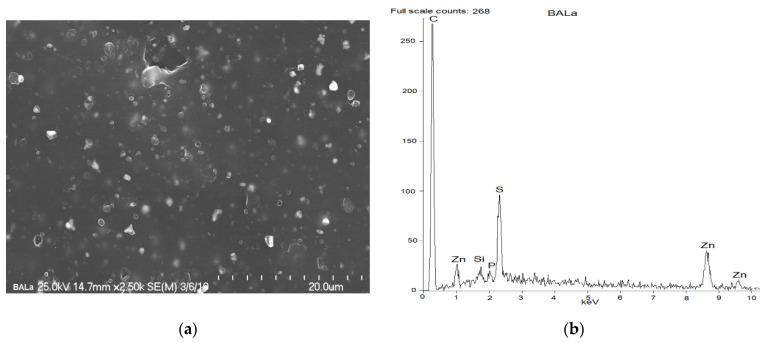
Dispersion of curatives in EPDM vulcanizate containing BALa: (**a**) SEM image; (**b**) EDS analysis.

**Figure 21:**
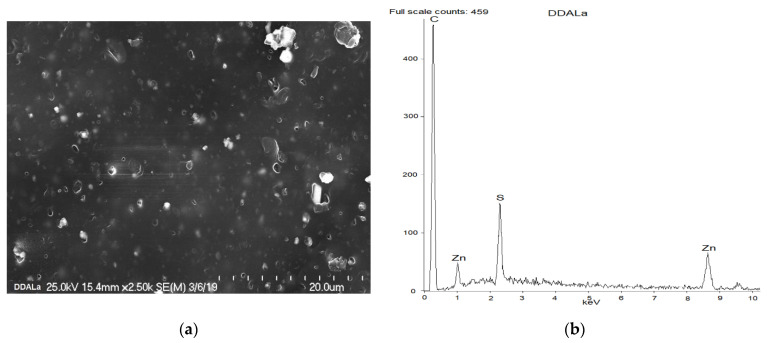
Dispersion of curatives in EPDM vulcanizate containing DDALa: (**a**) SEM image; (**b**) EDS analysis.

**Figure 22:**
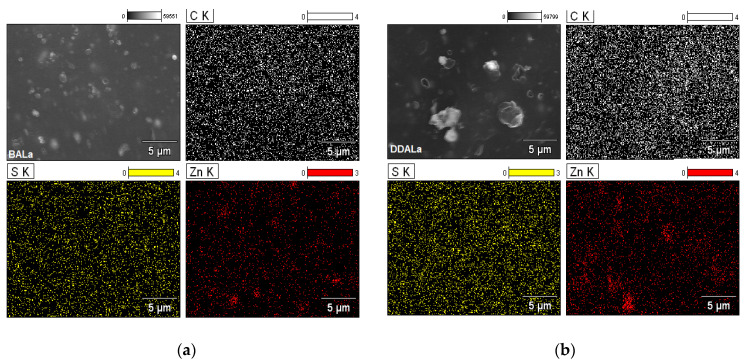
EDS maps for EPDM vulcanizate containing: (**a**) BALa; (**b**) DDALa.

As was already mentioned in the literature, TSILs, especially those bearing carboxyl functional group, are dedicated to dissolving metal oxides [22,23]. Therefore, TSILs were supposed to interact with ZnO used as the vulcanization activator, consequently increasing its solubility and facilitating its dispersion in the rubber matrix. In elastomer technology, homogenous dispersion of curatives and distribution of their particles in the elastomer matrix is very important aspect. Most solid components exhibit a high agglomeration ability in the elastomer matrix, so achieving a good interphase adhesion and homogenous distribution of the curatives is technologically difficult. The interaction between curatives in the elastomer matrix should be maximized to enhance the crosslinking efficiency and consequently the final crosslinking degree of the elastomer. Therefore, the influence of DDALa and BALa on the dispersion of ZnO and curatives particles was investigated. EPDM compounds with simplified compositions, i.e., containing only a curing system and an ionic liquid, were prepared and studied using SEM/EDS analysis. The content of additional ingredients, such as fillers, mineral oil, and CaO, may hinder the influence of the ionic liquid on the dispersion of curatives in the elastomer matrix. Results obtained for the vulcanizate without IL and with addition of TSILs are presented in Figure 19, Figure 20, Figure 21 and Figure 22.

SEM image showed that dispersion of the curative particles in the reference vulcanizate without addition of IL was inhomogeneous (Figure 19). The average size of created agglomerates was greater than 1 μm and these agglomerates were poorly wetted by the elastomer matrix. Bands for Zn and S elements in the EDS spectrum collected for this vulcanizate indicated that the agglomerates showed in the SEM image mainly consisted of ZnO and sulfur. This could also be ZnS, which is formed as a by-product during crosslinking reactions. From analysis of SEM images of the vulcanizates with TSILs (Figure 20 and Figure 21), the dispersion of curatives was quite homogeneous, especially for BALa (Figure 20), although it was more of a microdispersion than a nanodispersion. The clusters of particles were uniformly distributed in the EPDM matrix and well wetted by the elastomer. The bands for C, Zn and S elements were identified in the EDS spectra of the vulcanizates containing TSILs. To determine which components of the curing system have the greatest tendency to agglomerate in the elastomer matrix, EDS analysis was also presented in the form of maps. Examples of EDS maps for the vulcanizates containing BALa and DDALa are shown in Figure 22.

EDS maps collected for the vulcanizates containing BALa and DDALa showed that sulfur was quite homogeneously distributed in the elastomer matrix, whereas ZnO particles showed some ability for agglomeration, especially for DDALa; however, these agglomerates were quite uniformly distributed in the elastomer matrix.

### 3.7. Effect of TSILs on Cure Characteristics of EPDM Compounds and Crosslink Densities of Vulcanizates

Results of the studies described above confirmed that the carboxyl group of ionic liquid DDALa in the form of lactate anion reacted with ZnO in the range of vulcanization temperature of rubber compounds, consequently improving the dispersion of ZnO and curatives in the elastomer matrix. This should facilitate activation of the sulfur vulcanization. Thus, in the next part of research the effect of DDALa and BALa on the vulcanization parameters of EPDM compounds as well as the crosslink density and performance of EPDM vulcanizates was investigated.

**Figure 23:**
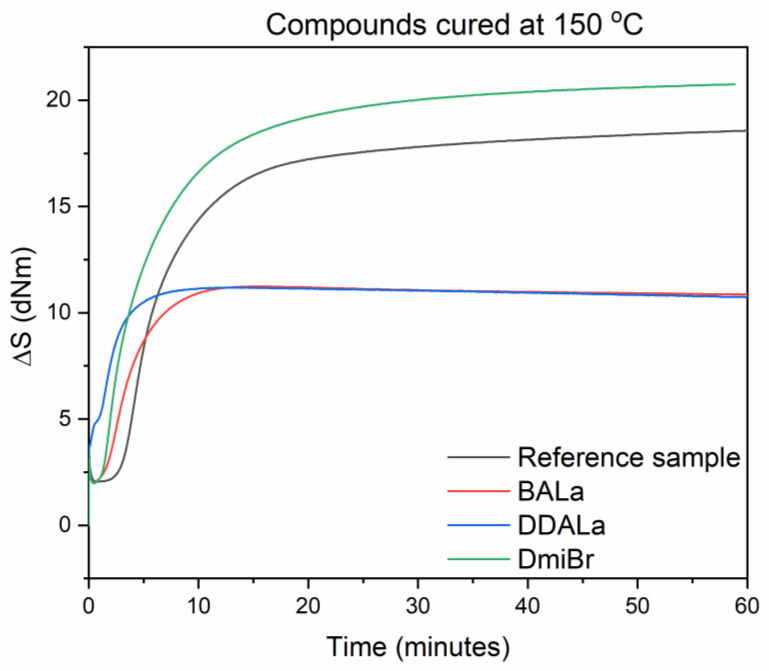
Rheometric curves at 150 °C for EPDM compounds containing TSILs or DmiBr.

Rheometric measurements were carried out to establish the activity of TSILs and DmiBr during the sulfur vulcanization of EPDM compounds. The influences of TSILs and DmiBr on the torque increase during vulcanization, which corresponds to the crosslink density, as well as on the scorch time and on the optimal vulcanization time of EPDM, was determined. Taking into account the technological and economic viewpoint, the vulcanization of rubber compounds should quickly proceed at the lowest possible temperature. Therefore, as with one of our previous works [34], the vulcanization temperature was reduced from the commercially used 180 to 150 °C. The rheometric curves are shown in Figure 23, whereas the cure characteristics of the EPDM compounds with TSILs and DmiBr are listed in Table 7.

Taking into account the measurement error, applying TSILs and DmiBr did not considerably affect the minimum torque during vulcanization nor the viscosity of the uncured rubber compounds, as compared to the reference sample without ionic liquid. Regarding the maximum torque and consequently the torque increment, the EPDM compounds containing TSILs exhibited significantly lower torque increases (approximately 9 dNm) than the reference sample (16.5 dNm), suggesting a lower crosslinking degree of the elastomer or plasticizing effect of TSILs. In contrast to TSILs, DmiBr had no detrimental effect on the ΔS (approximately 19 dNm) and, thus, had no effect on the crosslink density of EPDM elastomer. On the other hand, the performed studies showed that using TSILs with carboxyl groups, such as DDALa and BALa, allowed for a considerable reduction in t_95_ at 150 °C in comparison with t_95_ of the reference sample at the same temperature (Table 7) (from 29 min to 6 and 9 min for DDALa and BALa, respectively). The use of DmiBr allowed the t_95_ to shorten by only 3 min compared to the reference EPDM composite but the crosslink density was maintained at a similar level. However, reference vulcanizate without IL exhibited significantly higher crosslink density than vulcanizates with TSILs. The crosslink density of EPDM vulcanizate with BALa was comparable to the crosslink density of DDALa-containing vulcanizate. Thus, the reaction of ZnO with the lactate anion of TSILs had a complex effect on the vulcanization. It seemed to facilitate the activation of vulcanization and increase the rate of curing, as evidenced by a considerable reduction in t_95_ of EPDM compounds, but it caused a significant decrease in the crosslink density of the vulcanizates. The positive influence of the ZnO/TSILs reaction on the curing rate may result from the improvement of ZnO dispersion in the elastomer matrix. A homogeneous dispersion of ZnO increases the contact between its particles and other components of the curing system including vulcanization accelerators. It facilitates the formation of an active accelerator complex in the first step of the vulcanization, which then reacts with sulfur to form active sulfurating agent [24,42]. As a consequence, activation of the vulcanization and the process itself is easier and faster. On the other hand, it is commonly known that the content of ZnO in rubber compounds, and more precisely the amount of zinc ions available for interactions with curatives, strongly affects the crosslink density of the vulcanizates. According to Heideman et al., for ZnO loading below 5 phr, when the dispersion of its particles in the elastomer matrix was homogeneous, the crosslink density decreased with the content of ZnO in the rubber compound and the availability of zinc ions [42]. DSC analysis showed that ZnO reacted with the carboxyl groups of TSILs in the temperature range of 63–112 °C, which is lower than the vulcanization temperature (150 °C). The ionic interaction between the carboxyl groups of TSIL lactate anions and ZnO was confirmed by TOF-SIMS analysis, consuming some amount of ZnO decreasing the content of “pure” ZnO or reactivity of zinc ions and consequently the crosslink density of the vulcanizates. A similar reduction in the crosslink density of vulcanizates was observed when zinc ions were incorporated in rubber in the form of zinc stearate instead of pure ZnO [43]. As the performed studies did not show any reaction between ZnO and the cation or anion of DmiBr during crosslinking, no detrimental effect of the ionic liquid on the crosslink density of the vulcanizate was observed as compared with the reference sample.

TSIL-containing rubber compounds similar to the reference compound without IL, did not cure at 100 °C, which is important for their safe processing at this temperature. DmiBr reduced the safety of EPDM compounds processing at 100 °C compared to the benchmark without ionic liquid. Therefore, TSILs with lactate anion could be used when fast vulcanization of rubber compounds at 150 °C is required with safe processing at 100 °C.

### 3.8. Effect of TSILs on Temperature and Enthalpy of Vulcanization Studied Using DSC Analysis

Having investigated the influence of the BALa and DDALa on the cure characteristics of EPDM compounds, a DSC analysis was employed to study their influence on the temperature and enthalpy of EPDM vulcanization. The results for EPDM compounds are listed in Table 8.

Vulcanization of the EPDM compound without IL was a one-step exothermic process. The process occurred in a temperature range of 134–220 °C with an enthalpy of 4.8 J/g, resulting in the formation of sulfur bridges (C–S_x_–C) between macromolecules of rubber [44]. The studied TSILs, such as DDALa and BALa, significantly reduced the onset vulcanization temperature of EPDM (by approximately 41–47 °C) and increased the enthalpy of this process in comparison with the reference compound. This could result from the ability of DDALa and BALa for reaction with ZnO due to the presence of the carboxyl functional group. Consequently, this could lead to improved dispersion of the ZnO used as the vulcanization activator. Therefore, DSC analysis of rubber compounds proved that TSILs had a beneficial effect on vulcanization, allowing the temperature of this process to be reduced. DmiBr had a similar influence on the vulcanization temperature of rubber compounds, although the energetic effect of this process was lower as compared with the TSIL-containing EPDM.

### 3.9. Effect of TSILs on Tensile Properties and Hardness of EPDM Vulcanizates

The crosslink density is commonly known to considerablly affect the mechanical properties of the vulcanizates. TSILs significantly reduced the number of crosslinks in the elastomer network compared to the reference vulcanizate without the ionic liquid. Therefore, application of the studied TSILs can prevail the tensile properties and hardness of the vulcanizates. On the other hand, a positive effect on the vulcanization parameters could determine the potential application of these materials in the industry. Thus, the influence of BALa and DDALa on the mechanical properties and hardness of the EPDM vulcanizates was examined and the results are presented in Table 9.

Addition of TSILs with lactate anion (BALa and DDALa) significantly decreased the module at 100% elongation, increased the elongation at break (EB) and reduced the tensile strength (TS) by 2–3 MPa compared to the vulcanizate without IL. This was due to the considerably lower crosslink density of TSIL-containing vulcanizates. Moreover, the EB of the vulcanizate with BALa (581%) was about twice as large as for the vulcanizate without IL (321%), and the module at 100% elongation was approximately 2 MPa lower due than the lower crosslink density of the vulcanizate. However, it should be noticed that vulcanizates with DDALa and BALa exhibited quite comparable crosslink densities, whereas their EBs were very different (387% for DDALa and 581% for BALa, respectively). Thus, a plasticizing effect of BALa is also evident. It can also be seen that vulcanizates with DDALa and BALa were characterized by lower hardness as compared with the reference vulcanizate. Their hardness was approximately 10–11 ShA lower than that of the vulcanizate without IL due to the lower crosslink density. Despite lower hardness, the TSIL-containing vulcanizates can still be considered as a medium hardness rubber.

## 4. Conclusions

In this work, task-specific ILs (TSILs) with carboxyl groups dedicated for solubilizing metal oxides were examined. Results of DSC, TOF-SIMS and NMR analysis proved that, in contrast with alkylimidazolium IL, such as DmiBr, carboxyl group (lactate anion) of TSILs can react with ZnO during heating, which could improve the solubility of ZnO in the rubber and consequently cause better dispersion of the curatives in the elastomer matrix, resulting in a faster vulcanization of EPDM rubber compounds. Moreover, TG analysis proved that TSILs with lactate anions such as DDALa and BALa are thermally stable at the vulcanization temperature (150 °C) of rubber compounds and, thus, can be used to support the vulcanization.

BALa and DDALa allowed for considerable shortening of the optimal vulcanization time of EPDM compounds (from 29 min to 6 and 9 min for DDALa and BALa, respectively), while maintaining the safety of processing at 100 °C. Therefore, TSILs with lactate anions could be used when fast vulcanization of rubber compounds at 150 °C is required accompanied with safe processing at 100 °C. Furthermore, BALa and DDALa reduced the onset temperature of vulcanization (by about 41–77 °C) in comparison with the reference EPDM compound without IL.

Most importantly, despite a significantly lower crosslink density in comparison with the benchmark that does not contain an ionic liquid, EPDM vulcanizates with TSILs were characterized as having good mechanical performances and medium hardnesses, which allows for their potentially application. In addition, BALa showed a plasticizing effect, significantly increasing the flexibility of the vulcanizate, as evidenced by a significantly greater elongation at break than the vulcanizate with DALa, despite the comparable crosslink densities.

## Figures and Tables

**Table 1:** General composition of ethylene-propylene-diene monomer (EPDM) masterbatch, parts per hundred of rubber (phr).

Components, phr	Masterbatch
EPDM	100.0
Carbon black	125.0
Mineral oil	65.0
Chalk	40.0
CaO	7.5
ZnO	7.5
Stearic acid	1.5

**Table 2:** General composition of EPDM compounds containing Ionic Liquids (ILs) (phr); (MBTS, benzothiazole disulfide; Rhenogran TP 50, zinc dialkylditiophosphate; CBS, *N*-cyclohexyl-2-benzothiazolesulfenamide; TSIL, task-specific ionic liquid).

Components, phr	Reference Sample	Rubber Compounds with TSILs
Masterbatch	346.5	346.5
Sulfur	1.9	1.9
MBTS	1.0	1.0
Rhenogran TP 50	1.5	1.5
CBS	0.6	0.6
Stearic acid	1.5	1.5
TSIL	-	3.0

**Table 3:** General compositions of unfilled rubber compounds based on pure EPDM with ILs (phr).

Components, phr	Reference Sample	Rubber Compounds with Ionic Liquid
EPDM	100.0	100.0
Sulfur	1.5	1.5
MBTS	1.0	1.0
Rhenogran TP 50	1.5	1.5
CBS	0.6	0.6
Stearic acid	1.5	1.5
ZnO	7.5	7.5
TSIL	-	3.0

**Table 4:** DSC analysis for pure ZnO and its mixtures with ILs.

Sample	Temperature Range of Exothermic Peak (°C)	T_peak_ (°C)	∆H (J/g)
ZnO	-	-	-
DmiBr + ZnO	-	-	-
BALa + ZnO	81–112	90	3.6
DDALa + ZnO	63–109	92	4.2

**Table 5:** ^13^C NMR chemical shifts for pure DDALa and its mixture with ZnO (1:2).

Carbon	Chemical Shift (ppm)	
	DDALa	DDALa + ZnO
C1	14.4	14.4
C2	22.1	22.1
C3	31.8	31.8
C4	28.9	28.9
C5	29.4	29.4
C6	29.3	29.3
C7	29.1	29.1
C8	26.2	26.2
C9	22.6	22.6
C10	63.3	63.3
C11	50.4	50.4
C12	21.9	21.9
C13	67.3	67.4
C14	177.2	177.4
DMSO	40.0	40.0

**Table 6:** ^13^C NMR chemical shifts for pure DmiBr and its mixture with ZnO (1:1).

Carbon	Chemical Shift (ppm)	
	DmiBr	DmiBr + ZnO
C1	13.7	13.7
C2	21.8	21.8
C3	29.2	29.2
C4	28.6	28.6
C5	28.7	28.7
C6	28.4	28.4
C7	28.2	28.2
C8	25.3	25.3
C9	31.0	31.0
C10	48.5	48.5
C11	122.1	122.1
C12	123.3	123.3
C13	136.3	136.3
C14	35.6	35.6
DMSO	40.0	40.0

**Table 7:** Cure characteristics of EPDM compounds at 150 °C and crosslink densities of vulcanizates with ILs (ΔS, torque increase; t_05_, scorch time; t_95_, optimal vulcanization time; ν_e_, crosslink density; standard deviations: S_min_ ± 0.6, dNm ΔS ± 2.9 dNm, t_05_ ± 2.4 min, t_95_ ± 1.6 min, ν_e_ ± 0.9 × 10^−5^ mole/cm^3^).

EPDM Compounds	S_min_ (dNm)	S_max_ (dNm)	∆S (dNm)	t_05_ at 100 °C (min)	t_95_ (min)	ν_e_ (× 10^−5^ mole/cm^3^)
Reference sample	2.1	18.6	16.5	no curing	29	7.7
BALa	2.0	11.2	9.2	no curing	9	4.2
DDALa	3.0	11.2	8.2	no curing	6	4.7
DmiBr	2.0	20.8	18.8	28	26	7.7

**Table 8:** Temperature and enthalpy of EPDM vulcanization determined by DSC (standard deviations: temperature ± 17.0 °C; enthalpy (ΔH) ± 1.2 J/g).

EPDM Compounds	Temperature of Vulcanization (°C)	∆H (J/g)
Reference sample	134–220	4.8
BALa	93–196	6.0
DDALa	87–195	6.4
DmiBr	111–195	3.3

**Table 9:** Mechanical properties and hardness of EPDM vulcanizates with TSILs (SE_100_, modulus at a relative elongation of 100%; TS, tensile strength; EB, elongation at break; H, hardness).

EPDM Vulcanizates	SE_100_ (MPa)	TS (MPa)	EB (%)	H (ShA)
Reference sample	4.3 ± 0.1	10.9 ± 1.4	321 ± 57	70 ± 1
BALa	2.1 ± 0.1	8.1 ± 0.2	581 ± 34	59 ± 1
DDALa	2.9 ± 0.1	7.0 ± 0.2	387 ± 24	60 ± 1

## Data Availability

The data presented in this study are available on request from the corresponding author.
